# Social determinants of child mortality in Niger: Results from the 2012 National Verbal and Social Autopsy Study

**DOI:** 10.7189/jogh.06.010603

**Published:** 2016-06

**Authors:** Alain K Koffi, Abdou Maina, Asma Gali Yaroh, Oumarou Habi, Khaled Bensaïd, Henry D Kalter

**Affiliations:** 1 The Institute for International Programs, Johns Hopkins Bloomberg School of Public Health, Baltimore, MD, USA; 2 Department of International Health, Johns Hopkins Bloomberg School of Public Health, Baltimore, MD, USA; 3 Institut National de la Statistique, Niamey, Niger; 4 Ministry of Health, Niamey, Niger; 5 UNICEF/Niger country office, Niamey, Niger (retired staff)

## Abstract

**Background:**

Understanding the determinants of preventable deaths of children under the age of five is important for accelerated annual declines – even as countries achieve the UN’s Millennium Development Goals and the target date of 2015 has been reached. While research has documented the extent and nature of the overall rapid decline in child mortality in Niger, there is less clear evidence to provide insight into the contributors to such deaths. This issue is the central focus of this paper.

**Methods:**

We analyzed a nationally representative cross–sectional sample of 620 child deaths from the 2012 Niger Verbal Autopsy/Social Autopsy (VASA) Survey. We conducted a descriptive analysis of the data on preventive and curative care, guided by the coverage of proven indicators along the continuum of well child care and illness recognition and care–seeking for child illnesses encompassed by the BASICS/CDC Pathway to Survival model.

**Results:**

Six hundred twenty deaths of children (1–59 months of age) were confirmed from the VASA survey. The majority of these children lived in households with precarious socio–economic conditions. Among the 414 children whose fatal illnesses began at age 0–23 months, just 24.4% were appropriately fed. About 24% of children aged 12–59 months were fully immunized. Of 601 children tracked through the Pathway to Survival, 62.4% could reach the first health care provider after about 67 minutes travel time. Of the 306 children who left the first health care provider alive, 161 (52.6%) were not referred for further care nor received any home care recommendations, and just 19% were referred to a second provider. About 113 of the caregivers reported cost (35%), distance (35%) and lack of transport (30%) as constraints to care–seeking at a health facility.

**Conclusion:**

Despite Niger’s recent major achievements in reducing child mortality, the following determinants are crucial to continue building on the gains the country has made: improved socio–economic state of the poor in the country, investment in women’s education, adoption of the a law to prevent marriage of young girls before 18 years of age, and implementation of health programs that encourage breastfeeding and complementary feeding, immunization, illness recognition, prompt and appropriate care–seeking, and improved referral rates.

With a population over 15 million people in 2011, the Republic of Niger is West Africa’s second–largest country [1]. This landlocked country is characterized by chronic food security issues, natural crises, including droughts, floods and locust infestation, and a level of poverty that reflects more than a decade of periodic political instability. Niger’s poverty rate of 46.3% makes it one of the world’s poorest countries. Per capita income, at $360, puts it at the very bottom of the 187 countries ranked by the United Nations Development Program’s Human Development Index [2]. In this fragile nation, women and children suffer the greatest burden of poor health and inequality [3]. Niger’s social indicators have improved significantly over the past two decades, as progress toward the Millennium Development Goals (MDGs) is a main priority of the government.

The Government of Niger’s policies in support of universal access, provision of free health care for pregnant women and children, and strong nutrition programs have enabled the country to decrease child mortality at a pace that exceeded expectations. These policies are enshrined in general principles and international strategies such as primary health care and the Bamako Initiative. Thus, its health system is organized into three administrative and service levels: local/district, intermediary/regional and central/national. At the local level, public sector services are provided by community health posts (*Case de Santé*), integrated health centers (*Centres de Santé Intégrés*), and district hospitals. About 75% of health posts are staffed by CHWs (the rest by a nurse or midwife), and health centers and hospitals are staffed by at least one nurse, midwife or physician.

Recently, the Niger countdown case study showed far greater reductions in child mortality than in neighboring West African countries. In tandem with its efforts to tackle malnutrition, the government of Niger has put in place several measures to reduce childhood mortality. For the past few years, children under five have received free health care, while significant progress has been made in immunization coverage, recruitment of health staff and in the number of malaria cases treated. Collectively, these factors have contributed to a rapid reduction in the under–five child mortality rate, from 226 deaths per 1000 live births in 1995, to 128 deaths per 1000 in 2009 – a remarkable 43% reduction [4].

In preparing child mortality–reduction strategies in the post–2015 era, progress in reducing child deaths around the globe will require new and different strategies from those used to get the world to the current point. For instance, it is important for each country to know not only the magnitude of under–five mortality, but also the biological causes and social determinants of these deaths in order to assess needs and develop programs that will reduce avoidable child deaths more quickly. Thus, reliable direct estimates of the causes and the determinants of under–five deaths are needed to efficiently tailor evidence–based policies and programs.

A national verbal/social autopsy (VASA) study was conducted in Niger as part of the Child Health Epidemiology Reference Group’s (CHERG) recent efforts to directly measure the causes and determinants of neonatal and child mortality in selected high–priority countries.

The current paper aims to complement the recently published verbal autopsy findings [5] and reports on the social autopsy data of post–neonatal deaths. The objective is to provide insights into modifiable family, household, and health system factors that contributed to the deaths of children (1–59 months) from 2007 to 2010 in Niger, information that will be vital to health policymakers in government and non–governmental organizations as they develop new policies and programs for better resource planning in the post–2015 period.

## METHODS

### Study sample/VASA instruments/Data collection

Details of the statistical sample size calculation, VASA instruments, and data collection are available elsewhere [5]. In summary, the sample of deaths included in the Niger VASA study was identified by the Niger National Mortality Survey (NNMS) conducted in July–August 2010. The VASA study aimed to examine samples of the most recent 605 neonatal (0 to 27 days old) and 605 child (1 to 59 months old) deaths. The final VASA sample consisted of 1166 (96.4%) completed interviews of 1210 attempted, including 93 stillbirths, 453 neonatal deaths and 620 child deaths, with mean interview recall periods of 3.5 years (range 2–5 years) for the neonatal deaths and 2.7 years (range 2–5 years) for the child deaths. The current study focuses solely on the deaths of the 620 children (1 to 59 months).

The VASA questionnaire blends the Population Health Metrics Research Consortium (PHMRC) VA questionnaire with the CHERG SA questionnaire [6]. The interviews were conducted in French and the two main languages of Niger, Haoussa and Zarma, using a CSProX [7] software application developed for the VASA study to assist interviewers to capture responses with minimal data entry errors in the field directly on netbook computers.

There were seven data collection teams, each led by a field supervisor. The interviewers were 12 female and eight male native speakers of Haoussa and/or Zarma. The teams completed the data collection in 55 days.

### Statistical analyses

A descriptive analysis was conducted of the data on preventive and curative care, guided by the coverage of key indicators along the continuum of normal well child care and illness recognition and care–seeking for child illnesses encompassed by the BASICS/CDC Pathway to Survival model [8–10]. The 2010 NNMS data require the use of cluster sample weights to obtain nationally representative estimates. Thus all of the results presented here are weighted in order to compensate for threats to external validity inherent to the sample selection approach [11].

The list and definitions of some operational variables used throughout this paper are in the **Online Supplementary Document(Online Supplementary Document)**.

All the interventions examined by this study have been shown to be efficacious and effective in promoting child survival and thus are included among the interventions examined by the Lives Saved (LiST) tool [10] or recommended by the WHO, and so should be accessible to all children.

The SA data also assessed factors that might help explain why desirable actions were not taken, including socioeconomic and demographic factors, recognition of illness severity, decision makers, and self–identified care–seeking constraining factors.

### Ethical approval

The study was approved by the National Consultative Ethics Committee of the Niger Ministry of Health and by the Institutional Review Board of the Johns Hopkins Bloomberg School of Public Health. All the study personnel received training in ethical principles and practices for human subject’s research, and informed consent was given by all study participants before the VASA interview was conducted.

## RESULTS

A high proportion (91.5%) of the respondents, who were selected to be the child’s main caretaker during the fatal illness, were the child’s mother.

### Socio–demographic characteristics of the deceased children (1–59 months) and their households

The sociodemographic characteristics of the deceased children are presented in **Table 1**. The median age at illness onset was 12 months (SD = 14.0; range 0–day to–48 months) and the median illness duration was 7 days (SD = 40.1; range 0–day to 10 months). The majority of deaths occurred in the post–neonatal (1–11 months of age) and second–year (12–23 months of age) periods, 44.5% and 21.8%, respectively. The data showed slight differentials between deaths of females and males, with a male ratio of 96. Most (71.8%) of the deceased children were born at home; the majority (n = 470 or 75.9%) also died at home. The vast majority (87.2%) of mothers did not have or could not present a vaccination card for the deceased child.

**Table 1:** General mortality indicators and demographic characteristics of 620 children (1–59 months) deaths, Niger, 2007–2010

Characteristics	Frequency (No.)	Percent
**Median age at illness onset (in months)**	12 (SD = 13.98)	
**Median illness duration (in days)**	7 (SD = 40.11)	
**Median age at death (in months):**	12 (SD = 13.86)	
1–11	276	44.5
12–23	135	21.8
24–59	209	33.7
**Sex:**		
Male	304	49.1
Female	316	50.9
**Masculinity ratio (Boy/Girl×100)**	96	
**Place of birth:**		
Hospital	22	3.5
Other health provider or facility	143	23.0
On route to a health provider or facility	8	1.4
Home	445	71.8
Other	2	0.4
**Place of death:**		
Hospital	52	8.3
Other health provider or facility	63	10.2
On route to a health provider or facility	24	3.9
Home	470	75.9
Other	10	1.6
Don’t know	1	0.1
**Child possessed a vaccination card:**		
Yes, seen	79	12.8
Yes, not seen	352	56.8
No card	189	30.4

SD – standard deviation

**Table 2** shows the characteristics of the mother, her domestic partner, and the household. Approximately 97% of the mothers were married or living with a man at the time of the interview; the vast majority (94%) entered into a union before 20 years of age and had little or no education. Indeed, 87.3% had 0 years of schooling. The occupation most cited for the father as the breadwinner was farmer/agricultural worker (70%). Average household size was 7.7 persons. Only 11% percent of the households had electricity, one in three had access to an improved source of drinking water, 7% used improved sanitation (flush or improved pit toilet) and 98% of the households used firewood for cooking. About 10% of the households had flooring made of cement and 32% had a separate room for cooking. It took on average 80 minutes for the caregiver to reach the usual health care center from her household. The families had been living in the same community for about 18 years on average, yet 61% of the mothers reported they did not have anyone to help them during their child’s illness.

**Table 2:** Characteristics of the mother and her household, 620 children (1–59 months) deaths, Niger, 2007–2010

Characteristics	Frequency (No.)		Percent
**Married or living with a man**	599		96.6
**Mean age when first married (years):**	15.7 (range 12–30)		
12–15	355		59.3
16–19	208		34.7
20–30	36		6.0
**Mother’s mean age at time of child death (in years):**	27.7 (range 11–50)		
11–19	73		11.8
20–24	149		24.0
25–29	152		24.5
30–34	103		16.7
35–50	109		17.6
Don’t know	34		5.6
**Mean years of maternal schooling:**	0.6 (range 0–10)		
0	541	87.3	
1–3	15	2.5	
4–6	26	4.3	
>6	22	3.5	
Don’t know	15	2.4	
**Father years of schooling (mean years of schooling):**	1.0 (range 0–16)		
0–3	499	80.5	
4–6	13	2.1	
>6	40	6.5	
Don’t know	35	5.7	
**Household characteristics**			
Main breadwinner:			
Father	596	96.2	
Mother	11	1.8	
Other	13	2.1	
Main breadwinner is farmer/ agricultural worker	435	70.2	
**Mean years continuously living in community**	17.7 (range 0–69)		
**Household size (mean)**	7.7 (range 2–25)		
**Household has electricity**	67	10.8	
**Use of piped water in–house water supply**	208	33.6	
**Use of improved sanitation (improved pit for toilet)**	43	7.0	
**Separate room for cooking**	198	32.0	
**Household uses firewood for cooking**	609	98.2	
**Floor of the house made of cement**	63	10.2	
**Mean travel time to nearest health facility (min)**	80.4 (range 0–1380)		
**Social capital:**			
In last 3 years, community worked together on at least 1 of the following: schools, health, jobs, credit, roads, public transport, water, sanitation, agriculture, justice, security, mosque/church	540	87.2	
Mother was NOT able to turn to any persons or community groups or organizations for help during the pregnancy or child’s fatal illness	379	61.2	
Mother and her family have never been denied any of the following community	536	86.4	

### Preventive home care

**Figure 1:**
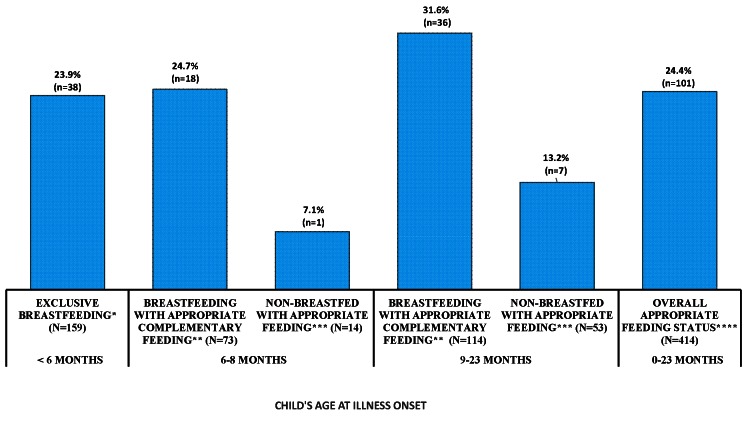
Breastfeeding and complementary or replacement feeding for children whose illness started at age 0-23 months. Legend: *Child’s illness began before 6 months of age (0-5 months), he/she was being breastfed at the time of fatal illness and was not given anything but breast milk as food. **Breastfed children whose fatal illness started at 6-8 months old and 9-23 months old who received, respectively, at least two and three complementary non-liquid feedings each day. ***Child's fatal illness started at 6-23 months old and he/she received at least four replacement feeds each day (including milk and solid, semi-solid and soft foods). ****Children whose fatal illness started at 0-23 months and satisfied either of the conditions above.

The exclusive breastfeeding and complementary feeding status among the 414 children whose fatal illnesses started between 0–23 months of age are presented in **Figure 1**. Overall, just 24.4% (n = 101) were appropriately fed. In more detail, about 23.9% (n = 38) of the 159 children whose fatal illnesses began at 0–5 months of age were exclusively breastfed. And only 28.9% (n = 54) of the 187 breastfed children 6–23 months old received the recommended complementary non–liquid feeds each day before the illness began.

**Figure 2:**
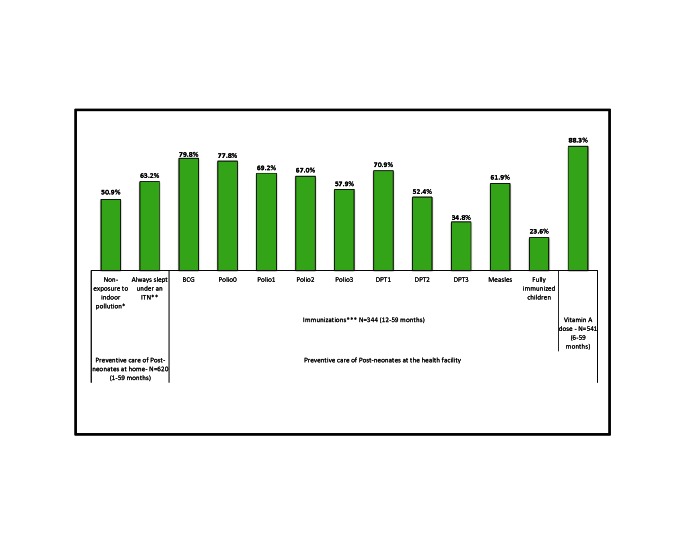
Coverage along the continuum of care for 1-59 month old child deaths in Niger from 2007-2010. Legend: *Proportion of children who were NOT usually nearby their mother when she cooked inside the home. **Insecticide-treated bed net. ***Information on immunizations was obtained either from the vaccination card or when there was no written record, from the respondent (mainly the mother). Polio0 is the Polio vaccination given at birth; fully Immunized children received BCG, measles, and three doses each of DPT and polio vaccine (excluding polio vaccine given at birth).

**Figure 2** shows the preventive home care received by the children along the continuum of care. About one in two (49.1%) of 405 children were likely to be exposed to smoke, ie, he/she was usually near the mother when she cooked inside the house. Sixty–three percent of the children always slept under an insecticide–treated bed net before their fatal illness began.

### Preventive health facility care

**Figure 2** further shows the percentage of the deceased children (12–59 months of age, n = 344) who received vaccinations against the six major preventable childhood diseases by one year of age. These findings were based on the vaccinations dates documented on the vaccinations cards, seen in about 13% of the cases, parental/respondent recall (87%), or a combination of the two. Overall less than a quarter (23.6%) of the deceased children 12–59 months were fully immunized against these diseases before their fatal illness began. The highest coverage was for BCG, DPT1 or PENTA1, and polio1, ranging from 69.2% to 70.9%. Sixty–two percent of children aged 12–59 months received measles vaccine. The deceased children were least likely to be fully immunized against DPT or PENTA by age one (just 34.8% had had all three doses).

Among the 12–59–month–old children, it took the group of fully immunized children on average 31 minutes less travel time to the nearest health care facility than the not–fully immunized children (63–minute vs 94–minute, *P* = 0.050).

### Curative care

**Figure 3:**
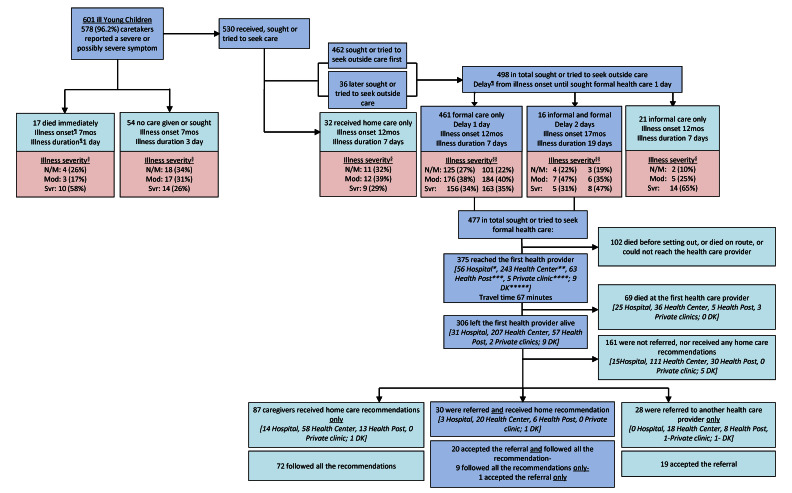
The “Pathway to Survival” for 601 deceased Young Children in Niger, from 2007-2010. Legend: §Illness severity at onset. §§Illness severity at onset and when caregiver decided to seek formal care. N/M=normal/mild, Mod=moderate, Svr=severe. *Hospital includes governmental or non-governmental hospital. **Health center includes governmental or non-governmental health center. ***Health post includes governmental or non-governmental health post. ****Private clinic formal or non-formal. *****DK: Don’t know where but with a community health worker, a nurse or a midwife.

**Figure 3** presents the breakdowns in the Pathway to Survival that contributed to the deaths of the children. Of the 620 completed interviews, 19 caretakers reported that they took some action at the time the fatal illness was noticed, yet the data on type of action was missing. Thus, the Pathway to Survival analysis included only the 601 children whose caretakers provided information on care–seeking.

Nearly all (96.2%) of caretakers of the 601 children recognized that their child had a severe or possibly severe symptom when they first noticed that the child was ill. More than 88% of the children (n = 530) received care, or their caretakers sought or tried to seek care; 17 (2.8%) children “died immediately”; and no care was given or sought for the other 54 (9.0%) children. About 58% of those who “died immediately” and 26% for whom “no care was given or sought” were ranked as being severely ill at the time their caregiver first noticed the illness. For these groups the fatal illness occurred at 7 months of age, and lasted, respectively, 1 and 3 days.

The vast majority (87.2%, n = 462) of the group of 530 children who received or whose caretakers sought or tried to seek care, first sought care outside the home; 68 (12.8%) first received home care, and 36 of these 68 later sought or tried to seek outside care. In total then, 498 received, sought or tried to seek care outside the home, and the median length (or delay) from the illness onset until formal health care–seeking was 1 day.

When care was sought outside of the home, the vast majority (92.6%, n = 461) received only formal care, 16 received both informal and formal care, and 21 received informal care only. The delay in seeking formal care was longer for those who sought informal and formal care than for those who sought only formal care (Median 2 days vs 1 day, *P* < 0.045).

Out of the 477 children for whom formal care was sought, 102 (21.4%) did not reach the health facility because they died before setting out, died en–route or could not reach the health care provider. The remaining 375 (78.6%) children reached the first health care provider after about 67 minutes travel time. About 2 in 3 (n = 243, 64.8%) of these 375 children went to a health center, 5 went to a private clinic, 63 to a health post, 56 (14.7%) went to a non–governmental organization (NGO) or governmental hospital, and 9 went to a health facility (staffed by a community health worker, a nurse or a midwife) for which the name or type could not be identified with the available data.

Sixty–nine (18.4%) of the 375 children that reached a first provider died at that provider, including the 25 out of 56 (44.6%) that reached a hospital. And 161 children out of the 306 (52.3%) that reached a health provider and left the provider alive were not referred nor given any home care recommendations. The remaining 145 were either only referred (n = 28) to a second health care provider, or only received home care recommendations (n = 87), or were referred and received home care recommendations (n = 30). In summary, just 58 (19.0%) of the 306 that left the first provider alive were referred. However, when recommendations were received, or referrals provided, most of the caregivers (67%–100%) followed all the recommendations or accepted the referral and went to a second health care provider.

**Figure 4:**
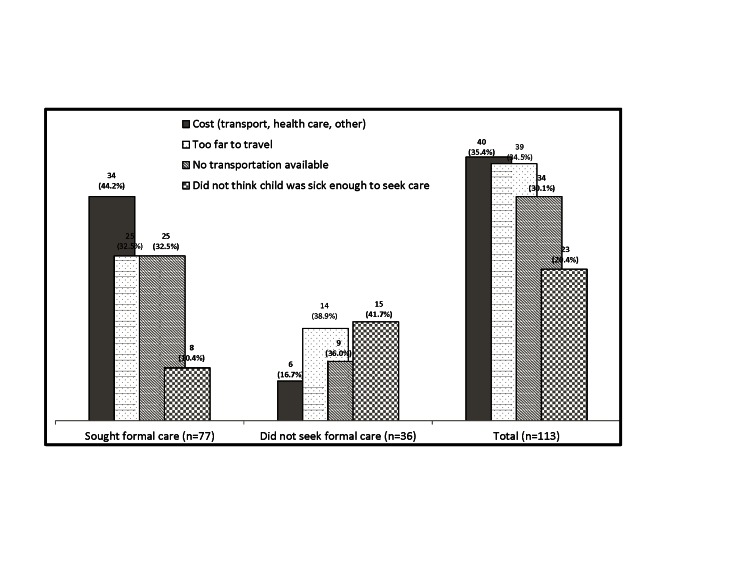
Main care-seeking constraints for child illness (N=113 caregivers).

**Figure 4** explores the care–seeking constraints for fatal child illnesses. In total, 113 caregivers reported that they had some concerns or problems in seeking care from a health care provider, for their child’s fatal illness. Cost (35.4%), distance (34.5%) and lack of transport (30.1%) were the primary disincentives for care–seeking at a health provider. Another constraint prevailed among the 36 children who did not seek any formal care: 41.7% of caregivers thought that the child was not sick enough to warrant care.

## DISCUSSION

The significance of this study lies in its goal to unveil the modifiable social, behavioral, and health system determinants of post–neonatal and child deaths in Niger between 2007 and 2010.

Findings from this study show that the majority of the deceased young children lived in households with precarious socioeconomic conditions, ie, lacking basic commodities such as electricity, sanitation, and clean water. There is a myriad of evidence to suggest that low standards of living adversely affect child morbidity and survival [12]. Improved safe water supply and community–wide sanitation are crucial interventions.

Exposure to household air pollution in the study setting was considerable: some 90% of the households lacked access to electricity and many relied on kerosene lamps and other polluting alternatives for lighting; about one in three children were usually near their mother while she was cooking indoors. Almost all of the households used biomass fuel for cooking. All of these conditions constitute a large and growing cause of environmental health risks, particularly among children due to the immaturity of their respiratory system. There is consistent evidence that exposure to indoor air pollution can lead to acute lower respiratory infections, and can increase the incidence of pneumonia to twice that of children not exposed [13,14]. Improving access to modern energy that emits less pollution, both in the home and the community, can benefit the health of children in the study setting.

Moreover, this study showed that both parents, especially mothers of deceased children had little or no education. Overwhelming evidence demonstrates the benefits of providing universal education to mothers. The children of mothers with no education are 2.7 times more likely to die than children of mothers who have more than 12 years of education [15].

This study showed that the vast majority of mothers of deceased children entered into marriage before their 20th birthday. The legal age of marriage for girls in Niger is 15 years. A law has been proposed to change the age to 18 years for girls but is yet to be adopted. It is known that early marriage has a direct impact on the health and mortality of children [16]. Understandably, the international community supports measures that aim to prevent early pregnancy and its poor health outcomes by preventing marriage before 18 years of age, by increasing knowledge and understanding of the importance of pregnancy prevention, by increasing the use of contraception and by preventing coerced sex [17].

The low rate of exclusive breastfeeding found in this study is of concern. The benefits of exclusive breastfeeding for 6 months include the reduction of the risk of diarrhea [18] and respiratory illness [19]. For the mother, exclusive breastfeeding is found to delay the return of fertility [20], and accelerate recovery of pre–pregnancy weight [21].

The overall high rate of undernourishment of children whose fatal illness began between 0–23 month–old revealed by this study is problematic. More striking, this study revealed that the vast majority of the non–breastfed children did not receive the daily appropriate recommended feeds. A previous study estimated that a non–breastfed child is 10 times more likely to die from diarrhea in the first six months of life than an exclusively breastfed child [22].

Undernourishment among children leads to malnutrition that in turn affects their immune system. They are more likely to become sick with common illnesses such as malaria, diarrhea, or respiratory infections, and the risk of death is very high [23]. Overall, it is estimated that nutrition–related factors contribute to more than one–third of deaths in children under five years of age [24].

Food taboos and lack of knowledge have been identified as the underlying causes of malnutrition in Niger [25] and are a hindrance when it comes to improving children’s health and nutritional status. Awareness–raising among families and communities must be one of the pillars to generate behavioral change in the population in general and among mothers of children under five in particular. More specifically, promotion of exclusive breastfeeding and complementary feeding practices has been proven to improve the nutrition and health of children and mothers [19–21,26].

The current study revealed that just 23.6% of deceased children 12–59 months were fully immunized before the fatal illness began, compared to 37.5% among alive children by 1 year of age as reported by the 2012 Nigerien demographic and health survey [27]. These low proportions suggest that the need to increase the vaccination coverage within the country cannot be overemphasized.

The study also revealed that the average travel time to the nearest health care facility inversely affected the immunization status of the deceased children. This finding corroborates those of previous studies [28].

Caregivers’ ability to recognize the illness and subsequently seek appropriate care for their children is pivotal to control diseases such as malaria, pneumonia, and diarrhea [29]. In the current study, caregivers were questioned only on illness signs and symptoms, but not on their recognition of specific illnesses as seen elsewhere [30]. And while almost all caregivers could report on a severe or possibly severe symptom or sign at the onset of their child’s illness, nearly one–fifth did not seek or try to seek care outside of the home. This finding suggests that the mothers failed to recognize the meaning of these signs of common severe childhood illnesses as shown in previous studies [31,32]; therefore many did not seek appropriate care for their children. This echoes the need for the Nigerien government to effectively implement and support the integrated community case management (iCCM) strategy that enables CHWs to provide life–saving interventions closer to home to address common childhood illnesses that were formerly provided only by facility based nurses and doctors [33]. This strategy is expected to result in a greater reduction of childhood deaths, especially if properly delivered at no cost to the country’s most disadvantaged communities.

In this study, nearly two–thirds of the children reached a first health care provider, offering the opportunity for effective treatment of the illness or referral if needed. Notably, 63 (16.8%) and 243 (64.8%) of 375 children that reached a first health provider were seen at a health post or health center, respectively. Health posts are able to manage uncomplicated childhood malaria, pneumonia and diarrhea, while providers at health centers treat all types of childhood illnesses and perform some laboratory tests and procedures such as lumbar puncture. Yet, these facilities have at their disposal only a minimal number of materials and drugs [34]. Therefore, providers at those facilities are entitled to refer severe or difficult cases to a higher level of care, usually to a health center, mini–hospital or a nearby district hospital [34].

The management of the children’s illnesses at the first provider could have been questionable. Despite that almost all the children were reported by their caregivers to have exhibited signs of severe or possibly severe illness, more than half who reached and left the first health care provider alive were not referred nor received any home care recommendations, suggesting a poor quality of care. The reasons why this significant proportion of sick children was not referred nor received any home care recommendation are unclear and warrant further study. It has been previously reported that Niger has a very low hospitalization rate due to a low referral rate and major accessibility problems [35], and that health care workers often do not refer, and caretakers frequently do not follow referral recommendations [32]. Healthcare workers may also have difficulty in complying with guidelines for referral – especially in rural areas where caretakers may be faced with many communication and transportation barriers [35]. Notably, cost, distance and lack of transport were reported by caregivers as the most important constraints to seeking care from a health provider during the child’s fatal illness, followed by not understanding the severity of the child’s illness, which was actually the main reason that constrained the caregivers who did not go to a health care provider for their child’s fatal illness.

This study has some limitations that were partly discussed in previous papers [5,36]. The long recall period of up to five years was mainly due to the retrospective design adopted for the VASA studies and the importance to include an adequate sample size of deaths. Consequently, this could have compromised the respondents’ recall of events, thereby, the validity of the findings.

In addition, while we sought some information from well–child and medical records available in the home, these were rarely available. For example, the majority of immunization records came from parental recall, and infrequently from the vaccination cards or health records. Nevertheless, several problems have been reported for the information provided both by vaccination cards and parental recall [37,38]. Parental recall may be inaccurate if parents forget the type and the numbers of vaccinations received, provide socially desirable responses, are not the person who brought the child to the vaccination session, or received incorrect information on vaccine schedules from providers. Vaccination cards may be incomplete or inaccurate if providers fail to record the doses administered or caregivers forget to bring the card to a vaccination session or, plausibly, unavailable if they were buried along with the deceased child.

Finally, a group of survivors would have allowed the analysis to test whether or not there were significant differences between the coverage of interventions among cases (deceased children) and controls (alive children). However, the lack of a comparison group in SA studies is common and not so necessary since we are studying interventions that should be accessible to all children.

## CONCLUSION

As governments and UN institutions work towards agreeing on a post–2015 framework, it is equally imperative that countries such as Niger commit explicitly to completing the job started by the MDGs, by adopting a target date to end preventable child deaths. The current study is timely in that it provided information previously not available in Niger either at the local or national level.

Of note, the government of Niger and stakeholders are engaged in steps for the use of the overall VASA study results to support the revision of the child survival strategy for the country. For example, in order to improve access to better health services for children, the government approved the transformation of some health posts into health centers with an average of 50 health posts transformed per year by December 2014; and the immunization program is being strengthened with the introduction and widespread provision of the Pneumococcal vaccine in the country [39].

Yet, the country needs to do more by adopting and enforcing the law to prevent marriage of young girls before 18 years of age, and encouraging women’s education and empowerment. Implementation of health programs that encourage breastfeeding and complementary feeding, illness recognition, prompt and appropriate care–seeking and improved referral rates for severe or possibly severe child illnesses will also go a long way towards curtailing child mortality in Niger.
