# Supplementary material for: Social determinants of child mortality in Niger: Results from the 2012 National Verbal and Social Autopsy Study
Source: J Glob Health. 2016 Feb 20;6(1):010603. doi: 10.7189/jogh.06.010603 (PMC4766790; doi:10.7189/jogh.06.010603)
Supplement: Online Supplementary Document [file jogh-06-010603-s001.pdf]

Online Supplementary Document

Koffi et al. Social determinants of child mortality in Niger: Results from the 2012 National Verbal and Social Autopsy Study

JoGH 2016;6:010603

OPERATIONAL VARIABLES DEFINITIONS

Non-exposure to indoor pollution: indicates the proportion of children who were NOT usually nearby their mother when she cooked inside the home.

Use of insecticide-treated bed nets for children: corresponds to the proportion of children that always slept under an insecticide-treated bed net before their fatal illness began.

Appropriate feeding status: identifies the proportion of children whose fatal illness started at 0-23 months and satisfied either of the conditions below:

(i) Exclusive breastfeeding: the child' illness began before 6 months of age (0-5 months), he/she was being breastfed at the time of fatal illness and was not given anything but breast milk as food; or (ii) Continued breastfeeding and appropriate complementary feeding starting at 6 months, i.e., breastfed children whose fatal illness started at 6-8 months old and 9-23 months old who received, respectively, at least two and three complementary non-liquid feedings each day; (iii) Appropriate replacement feeding for non-breastfed infants whose fatal illness started at 6-23 months old and who received at least four complementary feeds each day (including milk and solid, semi-solid and soft foods).

Fully immunized children: The Government of Niger has adopted the World Health Organization (WHO) guidelines for vaccinating children through the Expanded Program on Immunization (EPI). Children are considered fully immunized when they have received a BCG vaccination against tuberculosis, three doses each of Pentavalent (DPT-HepB-Hib) and polio vaccines, and a measles vaccination by the age of 12 months. Information on vaccination coverage was obtained in two ways: from health cards and from mothers' verbal reports. All mothers were asked to show the interviewer the health cards where vaccination dates are recorded for all antigens received. If the card was available, the interviewer then recorded the dates of each vaccination from the cards onto the questionnaire. The mother was then asked whether the child had received other vaccinations that were not recorded on the card, and if so, they too were recorded. If a child never received a health card, or if the mother was unable to show the card to the interviewer, the child's vaccination information was based on the mother's recall. The mother was asked to recall whether the child had received BCG, polio, Pentavalent, measles, and yellow fever vaccines. If she indicated that the child had received the polio or pentavalent vaccines, she was asked about the number of doses that the child received. The results presented here are based on both health card information and, for those children without a card, information provided by the mother. Thus, in this paper, a child was fully immunized if he/she had received these eight vaccines: BCG, measles, and three doses each of DPT and polio vaccine (excluding polio vaccine given at birth) by the age of 12 months

*Vitamin A supplementation from 6 months of age***:** or the proportion aged 6 months and older who received one or more doses of Vitamin A.
